# Developing image analysis pipelines of whole-slide images: Pre- and post-processing

**DOI:** 10.1017/cts.2020.531

**Published:** 2020-08-27

**Authors:** Byron Smith, Meyke Hermsen, Elizabeth Lesser, Deepak Ravichandar, Walter Kremers

**Affiliations:** 1 Department of Health Sciences Research, Mayo Clinic, Rochester, MN, USA; 2 Department of Pathology, Radboud University Medical Center, Nijmegen, The Netherlands; 3 Department of Health Sciences Research, Mayo Clinic, Jacksonville, FL, USA; 4 Department of Management Engineering and Consulting, Mayo Clinic, Rochester, MN, USA; 5 William J. von Liebig Center for Transplantation and Clinical Regeneration, Mayo Clinic, Rochester, MN, USA

**Keywords:** Image analysis, data science, analysis pipeline, deep learning, pathology, computer vision

## Abstract

Deep learning has pushed the scope of digital pathology beyond simple digitization and telemedicine. The incorporation of these algorithms in routine workflow is on the horizon and maybe a disruptive technology, reducing processing time, and increasing detection of anomalies. While the newest computational methods enjoy much of the press, incorporating deep learning into standard laboratory workflow requires many more steps than simply training and testing a model. Image analysis using deep learning methods often requires substantial pre- and post-processing order to improve interpretation and prediction. Similar to any data processing pipeline, images must be prepared for modeling and the resultant predictions need further processing for interpretation. Examples include artifact detection, color normalization, image subsampling or tiling, removal of errant predictions, etc. Once processed, predictions are complicated by image file size – typically several gigabytes when unpacked. This forces images to be tiled, meaning that a series of subsamples from the whole-slide image (WSI) are used in modeling. Herein, we review many of these methods as they pertain to the analysis of biopsy slides and discuss the multitude of unique issues that are part of the analysis of very large images.

## Introduction

**Fig. 1.**
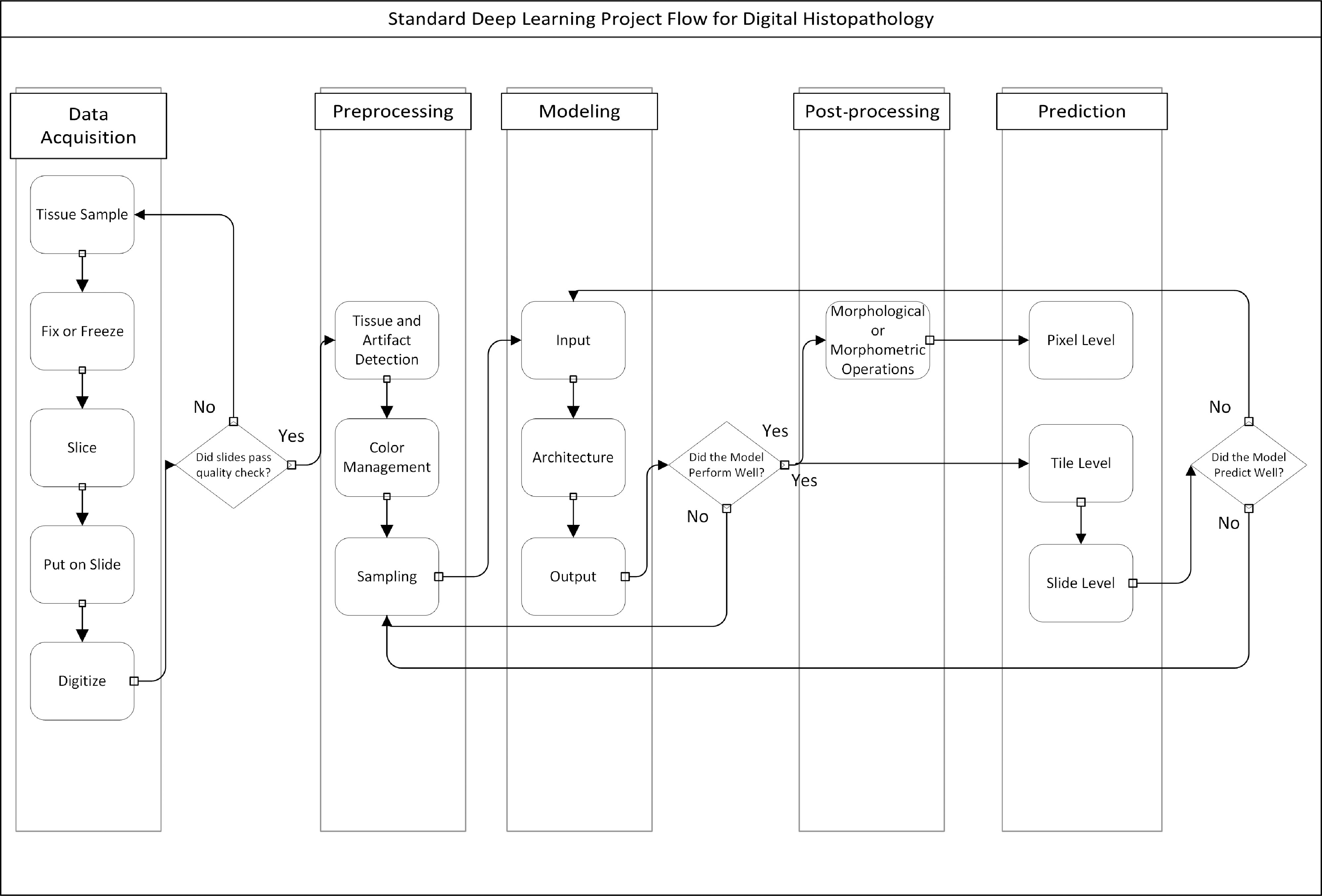
Typical flow of a WSI processing pipeline. In several instances, one may have to step backwards or start a stage over due to suboptimal outputs.

Recent developments in hardware and software have expanded the opportunities for modeling and analysis of whole-slide images (WSIs) in pathology. The possibility to train multi-layered (deep) neural networks combined with the generation of multiresolution images (i.e., WSIs) has generated entire new opportunities for the analysis of histopathologic tissue. Unfortunately, many of the resulting models lack a generalizable workflow from whole-slide preparation to results. This makes translation to clinical practice or application on large clinical trials a major challenge [1, 2]. Construction of end-to-end WSI analysis pipelines requires many more steps than model building in a controlled digital environment (Fig. 1). Tissue needs to be fixed, cut, stained, and digitized and the resultant images need to be “ cleaned” so that clinically relevant features can be selected and used in modeling. Because of the size of the images, subsamples of images or tiles are used for training deep learning algorithms. The raw output of the algorithm is post-processed and used to calculate possibly predictive data.

It is often said that 80% of an analyst’s time is spent on data cleaning rather than data analysis and image data is no exception. First, digitized images in pathology represent an overabundance of data (in terms of computational memory) and many of the pre- and post-processing steps focus on data reduction. Second, tissue preparation and digitization can cause substantial variability in a dataset. Analyzing images generated from a single center’s lab on an individual scanner may mitigate some of these issues, but variations may still exist across time as staining protocols and hardware changes. To combat these sources of variability, preprocessing and post-processing are used in tandem with complex modeling to create a reliable diagnostic tool (Fig. 1).

Once pre- and post-processed, aggregating predictions from the subsample (tile) level to the slide or patient level is another challenge as there may be multiple slides or tiles on a subject. Standard statistical tools, such as the mixed-effects model, that use random intercepts and slopes to account for multiple observations per patient, are not used in the context of deep learning although some alternatives have been considered [3–6]; these alternatives are described in more detail in the prediction section below.

Although many methods described in this article can and have been used in standard image analysis, we focus on how they augment the deep learning model development process. Specifically, we use the term deep learning to refer to the application of convolutional neural networks (CNNs) to images as opposed to other machine learning algorithms such as support vector machines or random forest predictions. To better understand this context, we briefly describe how deep learning is applied to images before moving on to the pre- and post-processing steps that improve output.

The exact steps used in the pre- and post-processing rely heavily on the end goal of any image analysis pipeline. Herein, we review a series of pre- and post-processing methods used in tandem with standard modeling techniques while motivating why some methods may be preferable to others. Available functions in R and Python are described throughout and summarized in Table 1 as a quick reference.

**Table 1.** A summary of select useful functions in image processing in Python and R

Program	Package/library	Function	Brief description
Python	Py_wsi	Sample_and_store_patches	Sample tiles (patches) from a WSI
	OpenCV	Dilate, erosion, etc.	
	scikit-image	Rgb2hed	Color deconvolution for WSIs
		Match_histograms	Histogram matching for color normalization
		Threshold_otsu	Otsu thresholding
	Scipy	Center_of_mass()	Calculate object location
		Find_objects()	Find objects in an array
		Label()	Label features in an array
	pgmagick		Image conversions
			
R	EBImage	computeFeatures	Calculate sizes of objects
		removeObjects	Remove objects
		Filter2D	Apply a filter to an image
		Dilate, erosion, etc.	Morphological operations
		Bwlabel()	Label objects
		Otsu()	Otsu thresholding
	Tiff, jpeg, png	Read/write	Read and write images of different file types
	Magick		Image conversions
	Reticulate		Compile Python code in R
Windows/Linux	ImageMagick		Command-line package for image manipulation

Note that a general recommendation is to start with the simplest solution and expand on this solution with increasing complexity; examples of this will be included throughout.

## A Brief Overview of Deep Learning for Image Analysis

Deep learning is defined as the use of deep neural networks for fully automatic discovery of patterns in (image) datasets. A specific type of network, CNNs are commonly used for the analysis of medical images throughout. In this article, we will focus on these types of networks. CNNs are “deep” in the sense that they follow a hierarchical analysis where the output of one “layer” of the network feeds into another layer and so on. In addition, these networks are differentiated from other hierarchical modeling methods (e.g., hierarchical regression models and multilayer perceptrons) in that they analyze an image by applying a filter to the image and feeding that filtered output to the next layer. These filters are used to identify local patterns. Filters are applied to an image by performing a convolution operation of the filter with the image. This effectively slides the filter across the pixels of the image, evaluating the product of the filter parameters with the image. Ultimately, this technique known as *parameter sharing* saves memory because a model is not applied to every pixel (such as a slope or parameter for logistic fit), but rather the filter parameters for each filter are shared across a neighborhood of pixels. Despite this, deep learning models used in image analysis can often range from 1 to 100 million parameters.

Another consequence of filtering is that it reduces the size of an image. One example is that a filter cannot sit on the edge of an image. Either the image has to be modified (i.e., padded) or the output will be smaller than the input. A filtering method known as pooling also reduces the image dimensions by taking windows of pixels and reducing them to a single-pixel output. As a result, output tile size post-filtering is almost always smaller than the input size. Depending on the model at use, this fact may necessitate the use of overlapping tiles when sampling from an image. More details are given below in the preprocessing section.

The main elements of a deep learning model are as follows:

1. *The input of the model* – Many images are too large to be analyzed directly and so sub-images are fed into the network. Because filtering an image can change the image size, model architecture can depend on input size.
2. *The architecture of the model* – The number of filters, the number of layers, and the overall connections from input to output.
3. *The output of the model* – Most commonly, a classification of the input into one or more classes or a segmentation of the image into separate classes (pixel-wise classification). Almost always the output will be of dimensions smaller than or equal to the input image.

As with any modeling processes, once these choices have been made the problem is converted to an optimization problem by defining a loss function to quantify how good or bad the fit was. A loss function is identified based on the endpoint being used and this is then optimized using an iterative approach. For example, the cross-entropy (binomial loss) is frequently used in classification as it would be used for any logistic model.

Just as with many machine learning methods, the hierarchical nature of deep learning models allows for complex relationships between input and output; however, this creates a sort of “blackbox” model that does not carry the same rigorous statistical background that a logistic model might. More specifically, this means that it can be challenging to understand how a single filter in a single layer may play a role in the overall model; there is no direct measure of association such as an odds ratio or *p*-value.

**Fig. 2.**
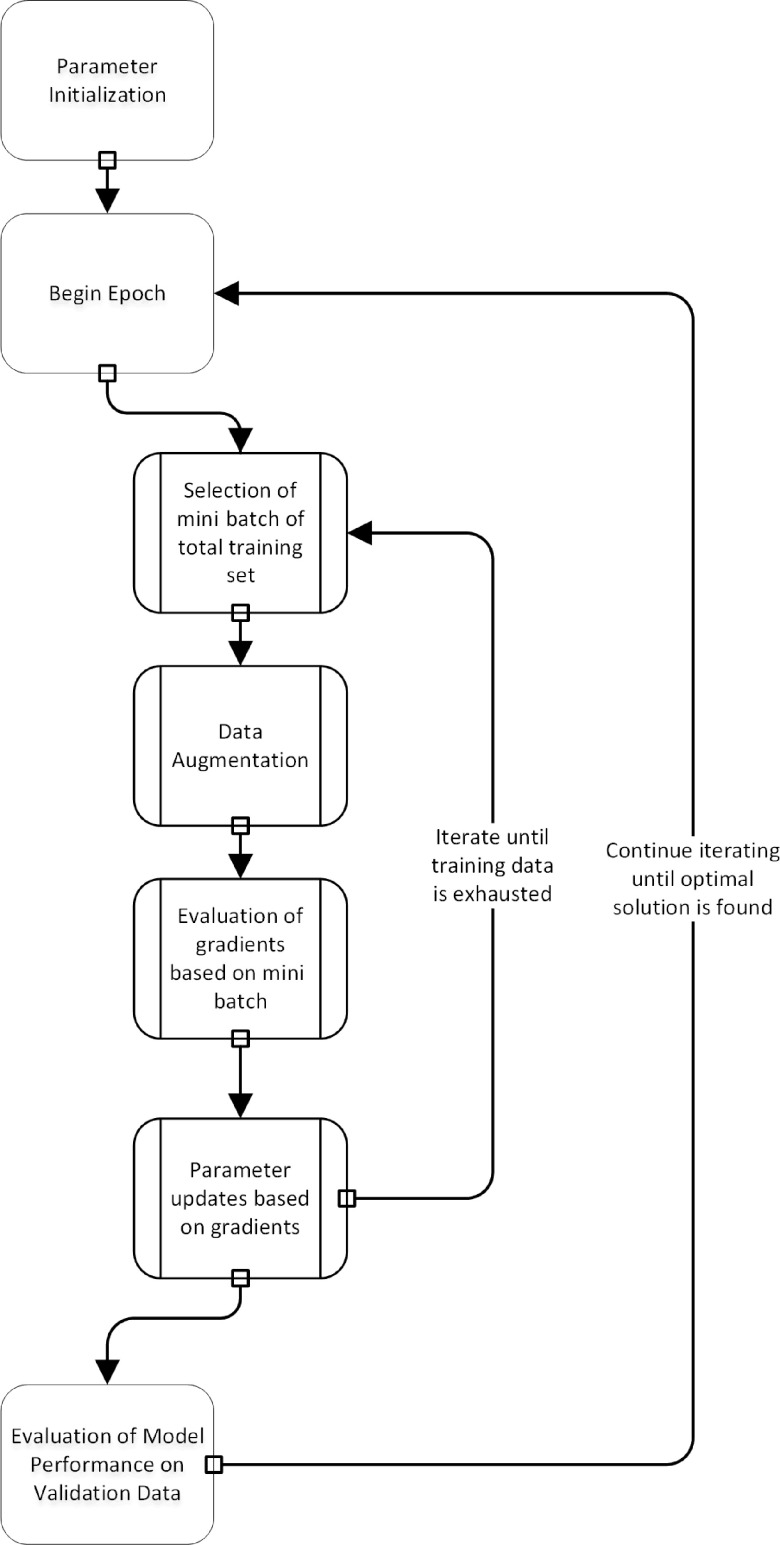
A flowchart of the training process in deep learning. Note that data augmentation is a step that is carried out during the training process.

For the development and testing of deep learning algorithms, the dataset is split into training, validation, and test sets. The network is optimized using the training and validation set, while the test set remains untouched. The optimization procedure in most deep learning environments is unique to its field (Fig. 2). The parameter settings are optimized iteratively, while the network aims to achieve better performance on the training set. Given that many CNN architectures have millions of parameters, modeling is prone to overfitting. To overcome this issue, it is a common practice to stop the training procedure when the performance of the model on the training and the validation set is optimized. This procedure is similar to optimization of hyperparameters in other machine learning algorithms where models are trained on the training set, hyper-parameter choices are evaluated on the validation set, and the final model performance is evaluated on the test set [7].

Another critical issue is that models must be robust to color shifts and geometric changes (translations and rotations). One way to build a model that is robust to these sources of variation is to apply the corresponding transformations during optimization. This is known as data augmentation.

Results of the deep learning algorithm may depend on each choice made during the model set up. Therefore, in order to find the best solution for a problem, many models are often fit with a series of different choices. This “guess and check” style is not unique to deep learning – frequently statistical analysis is iterative, based on model assumptions, checking interactions, model performance, etc. However, a standard model may run in seconds while deep learning models can take hours for a single optimization. Given that pre- and post-processing of output results may impact model performance, these steps act as additional choices that must be made in order to find an optimal solution. The typical flow of a project involving image analysis with deep learning can be seen in Fig. 1.

The most common packages for deep learning include the TensorFlow [8] and Keras [9] packages which are accessible in Python with analogues in R (although these require an installation of Python to run). Additionally, Caffe [10] and PyTorch [11] provide alternative frameworks for deep learning in Python.

## Data Acquisition

Before the development of an analysis pipeline is considered, one should explore the plethora of hardware that may be involved. The consequences of these choices can drive downstream methodology. First, there are several commercially available digital slide scanners and many of these scanners use unique image file formats [12]. While many efforts exist to format images as Digital Imaging and Communications in Medicine or another standard, similar to radiological images, no standard has been adopted [13, 14]. Just as with any image file type, there is a trade-off between image file size and resolution; image compression may save space, but ultimately impact final model performance by blurring features of interest. In the author’s experience, JPEG80 compression reduces space significantly while having minimal effect on image quality or network performance. Note that downstream modeling is based solely on pixel values stored in the form of arrays and, therefore, different file formats are more relevant to storage than analysis if the resolution is similar. Proper control of these sources of error can result in robust deep learning models that can process data across scanners regardless of file format [15].

The incorporation of graphical processing units (GPUs) into any analysis pipeline may be critical. While deep learning models necessitate the use of GPUs during the training process, predictions from these models can be performed in near real time on a standard workstation or computational processing unit (CPU). However, the repetitive calculations involved in most image analysis processes can be dramatically improved by parallelization and the inclusion of GPUs into any system used for high throughput pipelines. To facilitate these hardware dependencies, Griffin and Treanor recommends the inclusion of information technology (IT) teams in the construction process [2].

In addition to file size differences, color profile differences arise across laboratories and scanners. It is well known that differences in core facility staining procedures can give rise to high variability in image color profile, but so too can the digital scanning process [16]. The settings for all whole-slide scanners may determine the performance and/or generalizability of any downstream modeling, especially when no color augmentation was applied during the training of the model. One solution for this issue can be preprocessing to a standard color distribution, i.e., color normalization, which will be discussed below (preprocessing; color management) [17, 18].

## Preprocessing

The general purpose of preprocessing image data is threefold: (1) to discard data that is not informative or useful such as slide background or artifacts, (2) to create a consistent dataset, and (3) to enable processing of large WSIs during downstream modeling. The first stage is necessary to utilize computational resources in the most efficient way and to prevent generation of noise by, e.g., pen markings or dust particles. The second stage of preprocessing is used to reduce disturbing sources of image variability. The third stage is achieved by tissue sampling (dividing the image into tiles and feeding these batchwise to the network), because most deep learning models cannot process gigapixel images at once. Below, we provide more detail for each of these steps as well as suggestions for those beginning to develop image analysis pipelines.

An important note is that many of the methods mentioned within the post-processing section below may be useful in preprocessing although the motivations may differ. In preprocessing, morphological transformations are most often used to identify artifacts on the slide whereas during post-processing they are used to increase prediction interpretability.

### Artifact Detection and Tissue Segmentation

An important element in training and prediction is to limit the amount of non-informative data being analyzed. Non-informative data lies not just in the background of the slide, but also in artifacts on the slide that are not relevant to the histopathology of the individual patient. Artifact detection can be considered a quality control step in the image analysis pipeline as a way to prevent automated diagnostic errors based on uninterpretable image information. In addition, artifact removal allows downstream image normalization to proceed unhindered by pixel information that has no clinical relevance.

The myriad of artifacts that plague digital pathology remains a major hurdle in its application and these artifacts can arise during each stage of the data acquisition. At the slide preparation level, these often manifest as tissue fissures and folds, fingerprints, and watermarks. During digitization, several areas of focus are often declared in order to produce high-resolution WSIs. If these are sparse or disparate, image quality may change spatially causing focal artifacts or striations.

**Fig. 3.**
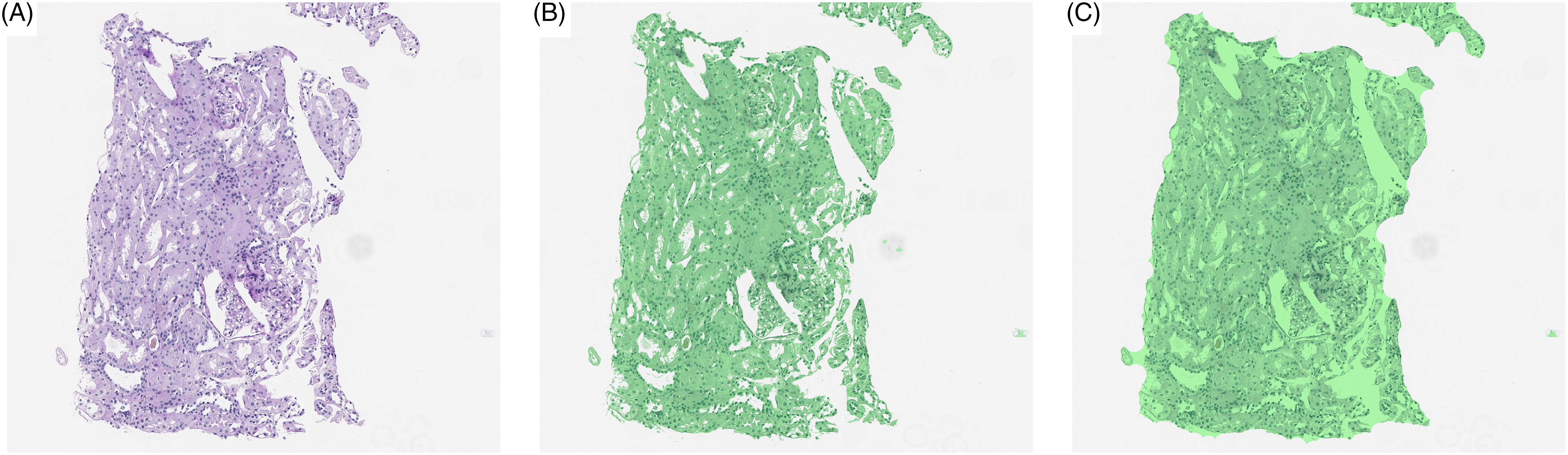
(A) Initial WSI, (B) tissue identified by thresholding (green), and (C) tissue identified by thresholding followed by filling hulls. By identifying tissue prior to analysis, slide background can be removed from the analysis.

Removal of these artifacts is frequently an important step in the downstream processing. A number of techniques exist to remove artifacts with a relatively simple implementation [19, 20]. One popular method focuses on Otsu thresholding [21] of the image which splits a grayscale image into “foreground” and “background” based on maximizing between-class variance (similar to clustering using the Ward’s minimum-variance method) or recursive partitioning trees (an example is given in Fig. 3B). From a quality control perspective, thresholding can be combined with a variety of other features such as edge detection filters or contrast measurements to check quality prior to analysis [19, 20].

The use of thresholds provides a very quick method to identify most artifacts, but not all. One elegant alternative is to use a deep learning segmentation algorithm to specifically discriminate background or noise (such as dust particles or pen markings) from foreground (tissue) [22].

### Color Management

**Fig. 4.**
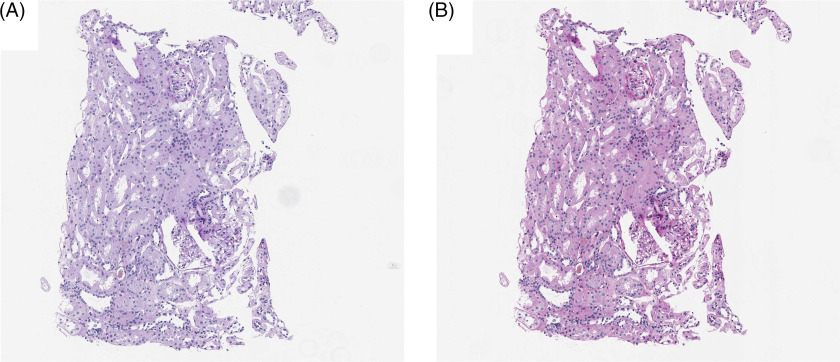
Color differences between an (A) Aperio ScanScope v1 and (B) Aperio AT2 slide scanner based on default color profiles using the same slide.

Preprocessing of images is critical to normalizing image data prior to downstream processing. While slide scanners capture the current state of a biopsy slide, the history of the slide can play a major role in the quality of the image. A common criticism of deep learning algorithms is that they do not generalize outside of an institution where the model is developed. Unfortunately, variations within a single institution may prevent generalizability as well. For example, an institution may decide to update its scanning hardware, possibly resulting in a change in color profile that may invalidate a trained model (Fig. 4).

More prevalent issues involve differences in stain intensity either from the staining process or fading over time. Staining very often varies from case to case even within the same lab, and dye fading is also often expected, but adjusting for these effects can be a challenge. The major issue is that histopathological dyes are often multicomponent and each component may fade at different rates. Furthermore, these dyes may fade differentially depending on which tissue is stained resulting in some features with higher variability than others.

Color management refers to standardizing color distributions across input images whether it is across sites or within a single center and has been tackled by many groups using various different methods [23]. As the vast majority of deep learning applications for digital pathology lying in the realm of cancer, color normalization has thus far focused on hematoxylin and eosin (H&E)-stained slides. One popular method presented by Macenko *et al.* introduces the concept of color deconvolution, i.e., separating hematoxylin from eosin [24]. They use a singular value decomposition (SVD) of a color space (hue, saturation, value) to establish color vectors that correspond to particular dyes. Unfortunately, a similar method may not generalize to a dye of three or more components because this no longer corresponds to a reduction in dimensionality relative to the color space. In order to overcome this limitation, methods have focused on color decomposition stratified by tissue type [25–27]. Clustering has also been proposed to identify the tissue type and perform color normalization within each cluster [28].

Extending the concept of tissue-specific normalization, a deep learning method has been adapted to recognize latent histologic structures and normalize them correspondingly using generative adversarial networks (GANs) [29, 30]. In a GAN, a generator network is trained to create “fakes” of input data (i.e., normalized slides) while an adversarial network identifies which images are true and which have been normalized. In this way, the two networks train each other, getting incrementally better at stain normalization so that the opposing network cannot distinguish between the true stains and normalized stains. This application allows for normalization that can be performed at the tile level and integrated seamlessly with downstream networks.

Many of the color normalization algorithms have been hand coded in languages such as Python and MATLAB, but could be adapted for use in R. K-means clustering and SVD are part of the base R package. K-means clustering is a standard clustering function contained within the scikit-learn library [31] for Python. Histogram matching from one image’s color distribution to another’s can be performed with scikit-image in Python.

During the training phase, exposing a model to as much color variation as possible can benefit the generalizability of the model. The easiest way to accomplish this is through data augmentation which should be the first step. However, for studies with larger sources of error, i.e., multicenter studies, further methods may be of value. Macenko’s method has been implemented in several programming languages and provides an overall color normalization that can be adopted quickly. Finally, if the color of a slide is tissue-specific, methods that normalize a slide uniformly rather than based on tissue composition may be of less utility. Here we recommend using more complex methods summarized above.

### Tissue Sampling

**Fig. 5.**
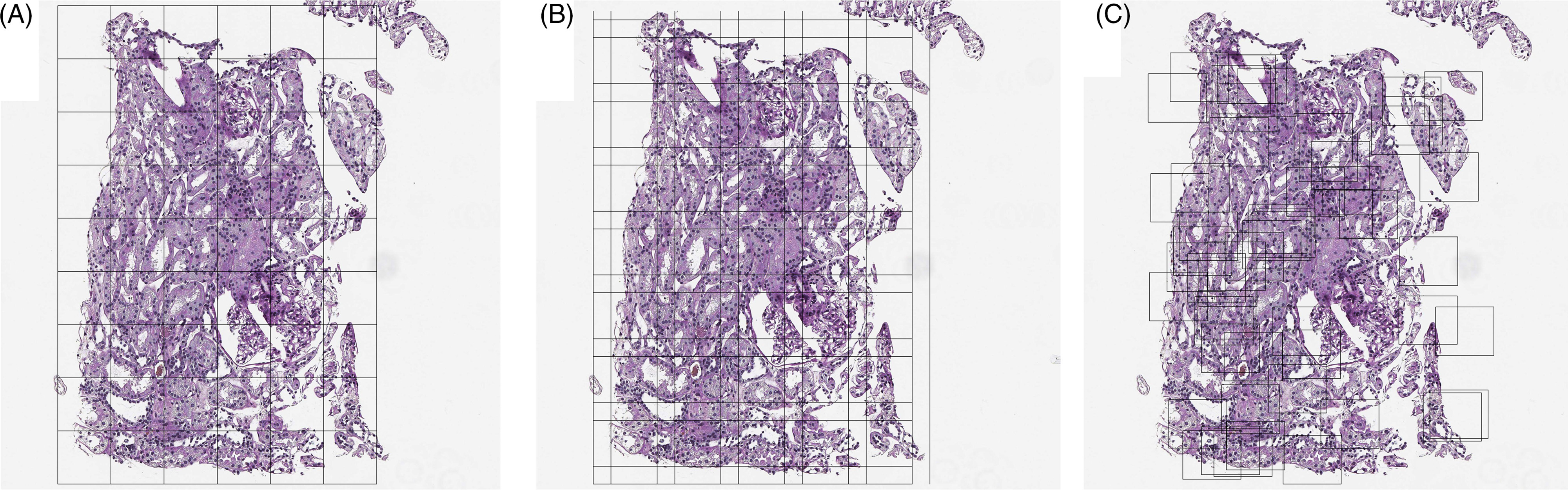
Tissue tiling based on (A) a regular grid, (B) an overlapping grid, and (C) random tiles.

The size of WSIs not only lead to computational limitations, but also necessitate additional steps in any analysis pipeline [12]. Most WSIs are on the gigapixel scale (on the order of 10,000 × 10,000 pixels) while the input to most deep learning algorithms analysis of images on the scale of 100-pixel squares (Fig. 5). This discrepancy typically means that training or prediction from a deep learning model requires tiling the image and reshaping these tiles into an array useable by the algorithms.

For the purpose of training, regions of interest (ROIs) are often annotated to curate a representative set of learning examples for the network. Unfortunately, these ROIs are often chosen to classify or segment ideal images, avoiding digital image flaws such as fingerprints, markers, digital striations, or background. Doing so may limit the generalizability of trained models to real-world datasets and clinical applications. Nevertheless, sampling in this way under samples slide background, sparing computational expense, and can be used in multistep analysis pipelines where an initial model is used to identify large-scale ROIs followed by analysis within those regions [22,32].

Sampling tiles from the ROI can be done in several ways (Fig. 5). First, a strict grid can be used although this may result in systematic sampling errors such as structure cleaving where certain patterns are cut into piecemeal. Overlapping grids can be used, but a choice in overlap amount is critical to balance redundancy and computational efficiency. Finally, random sampling to cover the region of interest can be used although this can result in high levels of redundancy as well. Image tiling can be performed automatically with the “py_wsi” package in Python.

For the purposes of prediction, random sampling is impractical to predict over the full region of interest. Overlapping, regular grids can be useful in the prediction of nominal class results. In the case of segmentation, this is often necessary because the output classification mask can be smaller than the input image. This is a direct result of the choice of using *zero padding* which adds zeros around the border of an image to assure that the convolution of a function within a neural network (filter) with the image results in an output of the same size.

Each decision during the training process determines the construct of the ground truth used to evaluate the loss function (likelihood) during optimization. Built into the loss function is the level in an image size hierarchy that is of interest for prediction or inference. In the case of classification, every tile can be given a label and this label may be chosen after the sampling process has been declared. Alternatively, the class can be declared at the slide level using the multiple instance learning (MIL) framework [33]. We discuss these choices more below. For segmentation, an image “mask” defines the class of each pixel and is sampled in accordance with the input image.

The final issue in sampling arises from the imbalance in class labels between good and bad pathology. This age-old statistical problem means that training sets are frequently constructed using a more balanced design to give networks exposure to bad pathology in sharp contrast to a general population. Alternatively, an unbalanced dataset may result in a lower sensitivity to identifying ROIs due to the fact that these regions may be confused with tissue that is not of interest due to nuances in the texture or color of the tissue. In order to overcome this, training can be followed by *hard negative mining* wherein false positive predictions are oversampled or added to the training set post hoc to prevent errors associated with balancing an unbalanced class.

## Post-Processing

**Fig. 6.**
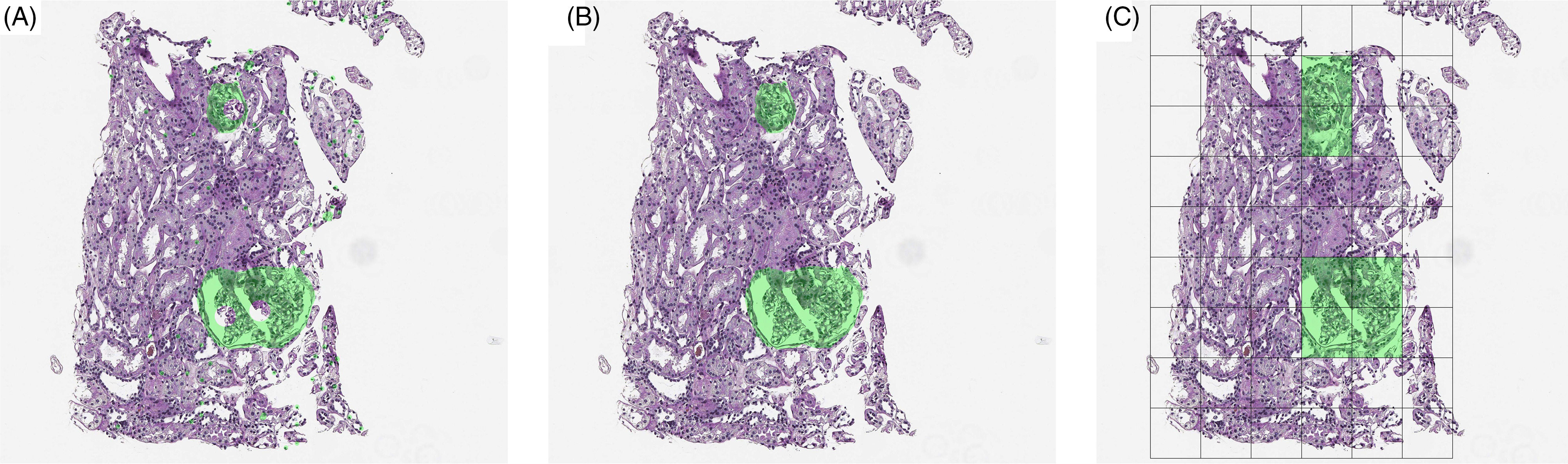
Hypothetical predictions of the presence of a glomerulus on a renal transplant biopsy from a deep learning algorithm either (A) by pixel (segmentation) prior to post-processing, (B) by pixel after post-processing, and (C) by tile. Note that raw output from a segmentation model may leave errant pixel predictions as well as hulls within objects of interest.

Many post-processing steps are more in line with the problem of segmentation rather than of classification. When predictions are at the pixel level rather than at the patch or WSI level, interpretability can be diminished by errant pixel predictions (Fig. 6A). This can occur as small areas of rare tissue types are often contained in large swaths of common tissue. Alternatively, several tissue subtypes may look similar and, therefore, any algorithm may be able to identify the larger structure, but fail to further characterize that structure. Post-processing, therefore, provides a set of tools to fix the smaller errors that a model makes.

In addition to errant pixel predictions, algorithms may predict gaps in an object (Fig. 6A). In order to improve interpretability, these gaps can be filled in using an operation known as “hull filling”.

Most post-processing steps are morphological in nature and have been reviewed in detail previously [34]. Available functions in R come chiefly through the “EBImage” package developed by Bioconductor [35] although the ImageR package offers very similar functionality (https://dahtah.github.io/imager/imager.html). A detailed set of examples is provided by Bioconductor as a vignette with code and sample data for many of the methods provided below (https://www.bioconductor.org/packages/release/bioc/vignettes/EBImage/inst/doc/EBImage-introduction.html). In Python, tools are available through the OpenCV [36] and SciPy packages [37].

### Hull Filling

A common occurrence in segmentation output is objects for which the circumferential predictions are of one class, but the interior predictions are of a different class. Intuitively, identification of the boundary of an object may be easier than identification of the interior. For this reason, the closed object prediction may form a “hull” (Fig. 6A). In order to improve the interpretability of segmentation output, hull filling is a quickly applied method of assign pixels in the interior of a hull to those of the bounding area. The hull-filling operation then converts all pixels within the closed object to the pixel classification that defines the closure (Fig. 6B). In order to perform this operation, pixels must be defined as a binary classification such as one versus all.

### Morphological Operations

**Fig. 7.**
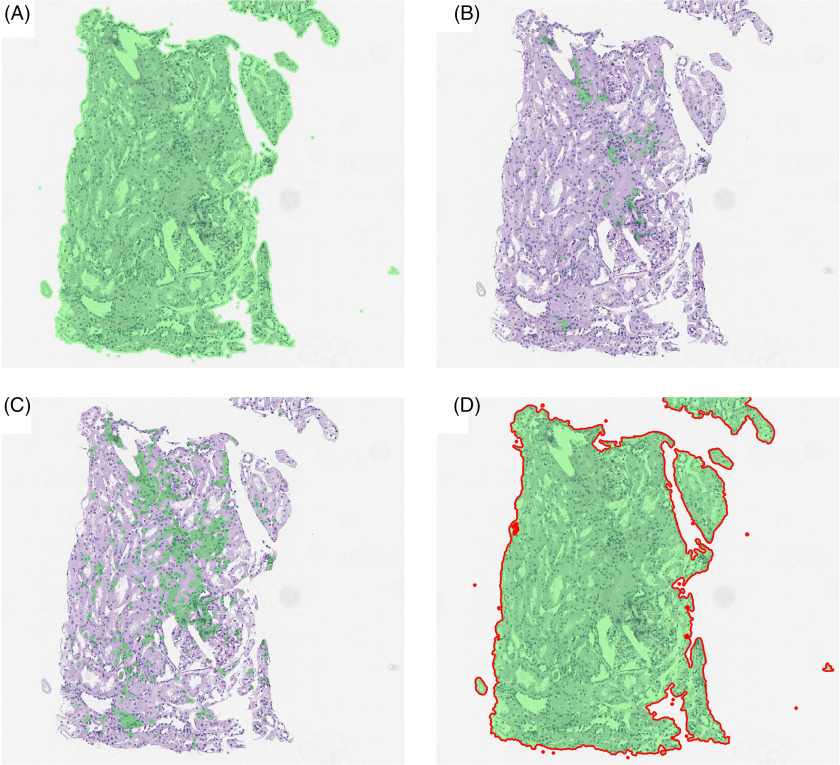
Morphological operations using an 11 × 11 filter size followed by hull filling. (A) Dilation, (B) Erosion, (C) Opening, and (D) Closing. Note that in this case, the dilation and closing with small differences in how close the segmentation is to the tissue boundaries.

Although hull filling can increase the interpretability of bounded objects, this does not overcome the fact that objects may be “open” with boundary pixels predicted less consistently than internal pixels of an object. To combat this issue, the morphological operations known as *dilation* and *erosion* may be used. These methods slide windows across the image, assigning the maximum (dilation) or minimum (erosion) pixel values within each window to the pixel at the center of the window, respectively. The resultant image can join disconnected boundaries due to the fact that they are within the same neighborhood of a pixel that defines the object. Using a dilation followed by an erosion results in a *closure* while the inverse results in an *opening*. This allows an analyst to programmatically join pixels of a similar type without expanding overall regions. Although these operations can be abstract, the difference in their results can be dramatic; a juxtaposition is provided in Fig. 7.

### Instance Segmentation

In medical images, it is often seen that structures lay close together, such as glands in prostate tissue and tubuli in kidney tissue. It can be useful to separate these structures after segmentation by a CNN to perform better post-processing or more accurate quantification. A way to identify individual objects is instance segmentation. With instance segmentation, the boundaries of an object are detected at pixel level by including an additional “boundary” class during training. This class can also be used to train a separate, binary network that is applied prior to the network aimed at the true structures of interest. The boundary class can subsequently be used to identify individual objects to which post-processing rules can then be applied. This method is more computationally expensive than simple segmentation and may provide more benefits for tightly-packed structures.

### Object Removal

Frequently small objects are identified errantly throughout a WSI. This can occur due to a variety of reasons such as random local patterns. A more challenging problem occurs due to the nature of slide preparation. Slides are prepared based on slicing tissue with a microtome, which means that objects of interest can be cleaved at their “tips” leaving little informative data, but a feature nonetheless. Prediction of these objects results in a true positive, but a clinical pathologist may deem the object too small to be used diagnostically. For these reasons it may be important to remove objects less than a specific size because they are likely to be incorrectly predicted or not useable in the clinical application.

The motivation behind these methods is to clean predictions that are not perfect. The exact choice in methods is generally unique to each image analysis project. Instead one should look at this set of methods as a toolbox where a combination of methods should be used to complete the cleaning task.

Object removal by object size requires a morphometric assessment of size for each object. The computeObjects() function in the EBImage package from Bioconductor [35] for R can quickly assess the size of every object identified in an image, but is limited in the scope of calculation in that it will calculate other features which may be less useful. The OpenCV [36] and SciPy [37] packages in python have similar functionality.

## Prediction

The tools used in any pipeline should be selected in the context of the final goals of the analysis pipeline. Much of the infrastructure in digital pathology is to augment the role of a pathologist, i.e., to make their decision-making process more accurate and timely. To this end, we present several scenarios similar to the work of Dimitriou *et al*. focusing on which level of experimental unit inference is being drawn: tile level or slide level [38]. In other words, is the researcher more interested drawn a conclusion about a small piece of the tissue or the slide as a whole?

Note that the majority of methods have been developed for classification although the concepts may be generalized to other endpoints by choice of the loss function during the model building process. In some cases, normal likelihoods (or other continuous measures) have been incorporated as well as partial likelihoods for survival analysis [6, 39]. Conventional analysis via deep learning is often not feasible due to image size.

Tiling an image results in multiple observations per slide. In general, the mixed-effects model which serves as the standard tool of statisticians in the case of multiple readings per experimental unit is not applied in deep learning. A standard log likelihood in the case of a mixed-effects model is a balance between regression components and variance components that are a combination of model error and random effects [40]. These are typically optimized using iterative methods such as Newton–Raphson stepping or the estimation–maximization (EM) algorithm [40].

Because the log likelihood is convex and differentiable convergence is theoretically guaranteed under gradient descent. However, estimates of the variance can be more challenging to incorporate into gradient descent algorithms using subsets of the data (mini batches) which are the gold standard in deep learning. This limitation renders the use of conventional mixed-effects modeling unusable. Instead, a close analogue known as MIL is frequently applied [33]. Below, we provide some examples of prediction at the tile level and then extensions to slide-level prediction similar to Dimitriou *et al* [38].

### Tile-Level Predictions

The simplest level of prediction is to treat each tile of the WSI as an independent entity (Fig. 6C). Tile-level predictions can be motivated by evidence of severity from even a single positive prediction. For example, a small region of cancer is still cancer at the slide level and patient level. However, some predictions may require a broader context which means that prediction at the tile level still requires information from other tiles or slides [41, 42]. An example is the CAMELYON17 challenge, where participants were asked not only to detect metastases in lymph nodes, but also to report whether this concerned isolated tumor cells, a micro-metastases, or macro-metastases on a patient level [43]. Only two participants actually measured the size of the largest region. More typically used measures were the number of detected metastases, mean detection size and standard deviation, mean detection likelihood, and standard deviation. Alternatively, the use of gradient boosting may provide a way to oversample borderline cases by weighting. This technique is more common in machine learning and utilizes sequential fits of a model followed by up-weighting of observations that were misclassified [17].

### Slide-Level Predictions

Due to the fact that the information contained within slides is dense, annotation of WSIs on a large scale has been a challenge. Data reduction is often required as a preprocessing step to focus on areas of interest. Popular strategies often involve one of two methods: (1) MIL [3, 44, 45] or (2) unsupervised methods with the goal of reducing the amount of input data prior to an analysis. MIL is chiefly used in classification problems by using “bags” (slides) of instances (tiles) to predict the class of a slide, iteratively selecting features from these tiles that would improve the classification. More specifically, a slide may be labeled as “positive” for some condition although the tiles composing the slide may be a mixture of positive and negative. An example would be declaring a full slide as cancer positive while only local regions of that slide (and therefore a few of the tiles belonging to the slide) as positive. On the other hand, a slide is only considered cancer negative if all tiles are considered negative. At each iteration of the modeling process, features of those tiles that result in a slide being declared positive are selected to improve the model. More details of these methods are provided in Carbonneau *et al* [33].

Unsupervised methods focus on two objects: (1) using a subset of the tiles to make a prediction over the full slide or (2) reduce the information contained within a single tile to a single value or a small set of values which could be used in downstream analysis. The methods range from more standard machine learning methods such as k-means clustering or principal component analysis (PCA) [5, 6]. Alternatively, deep learning has been applied to perform tile-level prediction followed by more standard analysis. Criticisms of these methods are that the two processes (tile compression followed by prediction) are performed independently and therefore improvements are iterative rather than simultaneous [38].

In an attempt to marry data reduction and prediction, several strategies have been proposed. The EM algorithm has been implemented to give more weight to slides that are more discriminatory when generating prediction estimates from these slides [44]. Noting that weighting is equivalent to differential sampling, Monte Carlo sampling has been implemented as well [4]. Simplifying tiles to single values as part of the modeling process has also been proposed [46].

## Discussion

The last decade’s evolutions in machine learning and whole-slide tissue scanners have changed the conversation around digital pathology and provided opportunities for increased accuracy and efficiency by the incorporation of computational pathology. While the promise of robust, novel models that are capable of assisting physicians in the decision-making process is exciting, the barrier to translate models to clinical practice cannot be overstated. Unlike conventional statistical models, image analysis frequently requires pre- and post-processing in order to achieve optimal solutions.

Digital pathology distinguishes itself from other medical image analysis scenarios in a large part due to the image size. Standard images, unpacked for the purpose of analysis, prevent full-scale modeling. This requires subsampling small tiles from a very large WSI, whether systematic using a grid or selecting at random.

The application of these systems can be complicated if the intent is to use modeling predictions in the framework of clinical trials. In order to meet regulatory demands, end-to-end system validation must be performed as each step creates a source of variation that could destabilize any results [47, 48]. While the Food and Drug Administration has taken a stance on this [49], the Digital Pathology Association (DPA) has provided detailed information on what steps need to be taken for validation [47].

Many of the methods in image pre- and post-processing may be familiar to statisticians such as data and dimensional reduction methods as well as linear and nonlinear transforms. However, some aspects of the process are unique to image processing. For example, the fact that image data is unstructured allows us to generate a nearly infinite number of predictors based on the intensity of a pixel’s intensity as well as the intensities of that pixel’s neighbors. Furthermore, most linear statistical models fit through the mean of the data. In this case, adding noise in an augmentation step does not impact predictions, but will inflate the model error. This may not be the case when implementing deep learning models that are highly nonlinear and hierarchical.

Throughout this manuscript, we provide a variety of options for processing images along with recommendations of where to begin. It is important to note that many image analysis projects are unique and our recommendations may not be valid or ideal. Trying different combinations of these tools will often be of benefit to any project. Also, note that the perspective taken for many of these processing steps is during the model training stage, but pre- and post-processing are just as useful in prediction.

Regardless of the methodology, analysis of WSIs is distinguished by unusually large datasets which are often analyzed piecemeal and clinically relevant predictions at the slide or patient level require some degree of reconstruction. While the standard methodology of mixed-effects modeling is unavailable, alternatives such as MIL provide a method to enforce similar conditions during the training process. By focusing on image cleaning, pre- and post-processing and predictive modeling, improvements in image classification and prediction are likely to aid the pathologist in improving the detection of important pathology and in reducing pathologist burden by automating many tasks.

## Data Deposit

Images from the manuscript, as well as the code used to create the images, have been uploaded here: https://github.com/BHSmith3/Image-Cleaning.
